# Population and pan-genomic analyses of *Staphylococcus pseudintermedius* identify geographic distinctions in accessory gene content and novel loci associated with AMR

**DOI:** 10.1128/aem.00010-25

**Published:** 2025-04-24

**Authors:** Jordan D. Zehr, Qi Sun, Kristina Ceres, Amy Merrill, Gregory H. Tyson, Olgica Ceric, Jake Guag, Sarah Pauley, Holly C. McQueary, Kelly Sams, Guillaume Reboul, Patrick K. Mitchell, Renee Anderson, Rebecca Franklin-Guild, Cassandra Guarino, Brittany D. Cronk, Claire R. Burbick, Rebecca Wolking, Laura Peak, Yan Zhang, Rebeccah McDowall, Aparna Krishnamurthy, Durda Slavic, Prabhjot Kaur Sekhon, David Needle, Robert Gibson, Casey Cazer, Jennifer Rodriguez, Beth Harris, Michael J. Stanhope, Laura B. Goodman

**Affiliations:** 1 Cornell University 5922, Ithaca, New York, USA; 2 Office of Applied Science, Center for Veterinary Medicine, US Food and Drug Administration 621252, Rockville, Maryland, USA; 3 Washington Animal Disease Diagnostic Laboratory, Washington State University 6760, Pullman, Washington, USA; 4 Louisiana Animal Disease Diagnostic Laboratory, School of Veterinary Medicine, Louisiana State University 70164, Baton Rouge, Louisiana, USA; 5 Ohio Department of Agriculture Animal Disease Diagnostic Laboratory 207837, Reynoldsburg, Ohio, USA; 6 Animal Health Laboratory, University of Guelph 3653, Guelph, Ontario, Canada; 7 South Dakota State University, Brookings, South Dakota, USA; 8 Veterinary Diagnostic Laboratory, University of New Hampshire 3067, Durham, New Hampshire, USA; 9 USDA APHIS National Animal Health Laboratory Network, Ames, Iowa, USA; Centers for Disease Control and Prevention, Atlanta, Georgia, USA

**Keywords:** antibiotic resistance, *Staphylococcus pseudintermedius*

## Abstract

*Staphylococcus pseudintermedius* is a common representative of the normal skin microbiota of dogs and cats but is also a causative agent of a variety of infections. Although primarily a canine/feline bacterium, recent studies suggest an expanded host range including humans. This paper details population genomic analyses of the largest yet assembled and sequenced collection of *S. pseudintermedius* isolates from across the USA and Canada and assesses these isolates within a larger global population genetic context. We then employ a pan-genome-wide association study analysis of over 1,700 *S*. *pseudintermedius* isolates from sick dogs and cats, covering the period 2017–2020, correlating loci at a genome-wide level, with *in vitro* susceptibility data for 23 different antibiotics. We find no evidence from either core genome phylogenies or accessory genome content for separate lineages colonizing cats or dogs. Some core genome geographic clustering was evident on a global scale, and accessory gene content was noticeably different between various regions, some of which could be linked to known antimicrobial resistance (AMR) loci for certain classes of antibiotics (e.g., aminoglycosides). Analysis of genes correlated with AMR was divided into different categories, depending on whether they were known resistance mechanisms, on a plasmid, or a putatively novel resistance mechanism on the chromosome. We discuss several novel chromosomal candidates for follow-up laboratory experimentation, including, for example, a bacteriocin (subtilosin), for which the same protein from *Bacillus subtilis* has been shown to be active against *Staphylococcus aureus* infections, and for which the operon, present in closely related *Staphylococcus* species, is absent in *S. aureus*.

**IMPORTANCE:**

*Staphylococcus pseudintermedius* is an important causative agent of a variety of canine and feline infections, with recent studies suggesting an expanded host range, including humans. This paper presents global population genomic data and analysis of the largest set yet sequenced for this organism, covering the USA and Canada as well as more globally. It also presents analysis of *in vitro* antibiotic susceptibility testing results for the North American (NA) isolates, as well as genetic analysis for the global set. We conduct a pan-genome-wide association study analysis of over 1,700 S. *pseudintermedius* isolates from sick dogs and cats from NA to correlate loci at a genome-wide level with the *in vitro* susceptibility data for 23 different antibiotics. We discuss several chromosomal loci arising from this analysis for follow-up laboratory experimentation. This study should provide insight regarding the development of novel molecular treatments for an organism of both veterinary and, increasingly, human medical concern.

## INTRODUCTION

*Staphylococcus pseudintermedius* is a coagulase-positive species (CoPS) within the *Staphylococcus intermedius* group, which also includes the three CoPS species, *Staphylococcus delphini*, *Staphylococcus intermedius*, *Staphylococcus cornubiensis*, and a coagulase-negative species (CoNS), *Staphylococcus ursi. S. pseudintermedius* is a component of the normal skin microbiota of dogs but is also an important causative agent of skin infections such as canine pyoderma and surgical wound infections, as well as ear and urinary tract infections (1). Carriage rates of *S. pseudintermedius* in non-canine animal species such as cats and horses are much lower but still linked to pyoderma in both these hosts (2–4).

Much of the recent research interest on *S. pseudintermedius* has centered on the identification of methicillin-resistant (MRSP) strains and the zoonotic potential of the species (1, 5, 6). MRSP strains have higher non-susceptibility rates to all antimicrobials compared to methicillin-susceptible *S. pseudintermedius* (MSSP) (7), and the overall presence of MRSP in humans is increasing (6). MSSP isolates are often also resistant to at least three classes of antimicrobials, that is, multidrug resistant (MDR). Calabro et al. (7) found 47% prevalence overall for MDR in a survey of 1,804 isolates tested by one US reference laboratory. In another recent study of *S. pseudintermedius* in the New England area, Bruce et al. (8) found that out of 171 isolates tested, 41 were MRSP, 130 were MSSP, and 80 were MDR. Such high and increasing levels of antibiotic resistance represent a significant current and future challenge for the veterinary community (9), as well as an important consideration in the treatment of human zoonotic infections (6).

Many genes have been identified from a wide range of bacterial species that are causatively linked to antimicrobial resistance (AMR), and these are cataloged on resistance databases such as the Comprehensive Antibiotic Resistance Database (10) and AMRFinderPlus (11). Despite progress in identifying AMR loci, many yet unidentified genes conferring resistance undoubtedly form the repertoire of resistance mechanisms in most bacteria species. A significant body of information is known about AMR mechanisms in the important human pathogen *Staphylococcus aureus* (reviewed in reference 12), and much of these loci are orthologous in *S. pseudintermedius* and are assumed to function similarly in dogs and cats. However, few studies have attempted to identify novel AMR mechanisms in *S. pseudintermedius*. One way to attempt to identify novel AMR loci is to correlate phenotypic susceptibility data with loci from genome sequence data and then form short lists of genes with significant correlations and follow up with laboratory experiments. Following on from the discovery that bacteria genomes comprise both core and accessory components (13, 14) and the resulting field of bacteria pan-genomics, genome-wide association study (GWAS) methods for bacteria were developed that use the gene presence/absence typical of bacteria accessory genomes as the variable for genetic correlation (pan-GWAS) (15). Many of the known resistance genes in *S. aureus* belong to the accessory genome (55%), and among these, 47 (27%) have been identified within the Staphylococcal Cassette Chromosome mec (SCCmec), which is responsible for resistance against various classes of antibiotics (16). The majority of these same or very similar SCCmec elements are carried by *S. pseudintermedius* (8, 17, 18). Based on work by our group (19) and others (20), current genomic-based prediction accuracy of the presence of known resistance loci (inclusive of resistance-determining mutations) is approximately 97%–98% depending on the antimicrobial. In addition to these known AMR loci, we hypothesize that a pan-GWAS approach could identify as yet unknown, putative resistance-determining genes from the *S. pseudintermedius* accessory genome. Herein, we employ such an unbiased pan-GWAS approach to correlate the presence and absence of *S. pseudintermedius* genes with resistance phenotypes in dogs or cats.

This paper details our examination of population genomics, covering the North American (NA) region in a global context, as well as levels of AMR in the largest comparative genomic data set yet assembled for *S. pseudintermedius*. We then go on to employ a pan-GWAS perspective on *S. pseudintermedius* isolates from sick dogs and cats collected as part of the US National Action Plan for Combating Antibiotic-Resistant Bacteria (https://www.hhs.gov/ash/advisory-committees/paccarb/working-groups/national-action-plan/index.html) and its surveillance for resistant bacteria in animals (21, 22). The analyzed collection includes *in vitro* susceptibility data for 23 different antibiotics from over 1,700 isolates across multiple regions of the USA and Canada, covering the period 2017–2020. We correlate loci, at a genome-wide level, with resistance/susceptibility data for this large set of antibiotics and shortlist encouraging loci for further experimental evaluation.

## MATERIALS AND METHODS

### Samples and genome sequencing

No research animals were used for this study. *S. pseudintermedius* cultures from dogs and cats in the USA and Canada were collected as part of two federal programs which monitor AMR profiles in animal pathogens routinely isolated by veterinary clinics and diagnostic laboratories. The US FDA Veterinary Laboratory Investigation and Response Network program (21) has a total of 30 participating animal diagnostic laboratories that save clinical (from a sick or deceased animal) *S. pseudintermedius* isolates from canine cases submitted each month by a veterinarian for diagnostic testing. Isolates were tested for *in vitro* susceptibility, cryopreserved in glycerol, anonymized of identifying information, and shipped to one of six assigned sequencing reference laboratories. Whole-genome shotgun sequencing was performed using Illumina chemistry (DNA Prep or Nextera XT library preparation) on the MiSeq platform. Genomes meeting standardized benchmarks (23) were uploaded to the National Center for Biotechnology Information (NCBI). The USDA APHIS NAHLN AMR Monitoring Program (24) has 36 participating laboratories saving both canine and feline diagnostic isolates. Isolates in this study were sequenced either by those laboratories or by the National Veterinary Services Laboratories, using the same methods and quality benchmarks.

Sample sources from the combined USDA-FDA surveillance set included skin or nailbed biopsies/swabs, wound/abscess swabs, ear swabs, respiratory fluid/tissue, urine, and blood, collected from 2017 to 2020 by five regional reference laboratories in Canada and 26 laboratories across the USA. All genome sequences from dogs and cats used for AMR pan-GWAS are available in the following NCBI BioProjects: PRJNA314609 and PRJNA510565.

### Antimicrobial susceptibility testing (AST)

*In vitro* antimicrobial susceptibility data were provided by the participating veterinary diagnostic laboratories with the Sensititre platform (Thermo Fisher Scientific) for automated broth microdilution using veterinary commercial panels. Interpretations of susceptibility were performed in February 2023 and standardized using the breakpoints provided in Table 1, based on a consensus of CLSI Vet01S (6th ed., February 2023). Antibiotics were interpreted independently to identify putative mechanisms associated with resistance unique to each antibiotic, in contrast to clinical determination of resistance to one antibiotic based on the result of another (i.e., cefazolin resistance inference based on oxacillin resistance). Samples that either came from an unknown infection source or did not have an MIC value provided were omitted. All AST data were based on broth microdilution with minimum inhibitory concentration (MIC); any isolates tested by disk diffusion were excluded. The proportion of resistant samples was compared between geographic regions with Pearson’s chi-squared tests using the prop.test function in the R stats package version 4.3.1, R version 4.3.1. If at least one proportion was significantly different from others (*P* < 0.05), pairwise comparisons were calculated with Bonferroni correction for multiple comparisons using the pairwise.prop.test function in the R stats package. Proportions were compared separately for samples resistant to greater than five antibiotic classes and samples resistant to three to five antibiotic classes (25). All MIC data are available in Excel format at https://www.fda.gov/animal-veterinary/national-antimicrobial-resistance-monitoring-system/integrated-reportssummaries.

**TABLE 1:** Breakpoints used for binary coding of susceptibility phenotypes (resistant above identified breakpoint)^*a*^

Antibiotic	Dog/cat	Human
Amikacin	≥16	
Amoxicillin/clavulanic acid	≥1	
Ampicillin	≥0.5	
Cefazolin	≥8	
Cefovecin	≥2	
Cefpodoxime	≥8	
Cephalothin	≥8	
Chloramphenicol		≥32
Clindamycin	≥4	
Doxycycline	≥0.5	
Enrofloxacin	≥4	
Erythromycin		≥8
Gentamicin		≥16
Marbofloxacin	≥4	
Minocycline	≥2	
Nitrofurantoin		≥128
Oxacillin		≥0.5
Penicillin		≥0.25
Pradofloxacin	≥2	
Rifampin		≥4
Tetracycline	≥1	
Trimethoprim-sulfamethoxazole		≥4
Vancomycin		≥32

^*a*^ Human breakpoints were used when neither dog nor cat breakpoints were available. MICs (μg/mL) meeting the reported breakpoint criteria for each antibiotic listed below are considered resistant, and otherwise are labeled as susceptible.

### Genome assembly, phylogenetic reconstruction, population genetics

Genomes not already assembled by NCBI were assembled using SKESA (26). Assemblies with >200 contiguous sequences (contigs) and outliers from a multi-dimensional scaling plot created from Panaroo v.1.2.9 (27) QC step were excluded from downstream analysis. All NA genomes were verified to have either dog or cat indicated in the host metadata. The final curated set of compiled dog/cat *S. pseudintermedius* genomes for the NA set was 1,746 (1,584 dogs and 162 cats). To examine where these NAm isolates fit within a broader population genetic perspective, an additional data set was compiled of global isolates from NCBI; this final set included 2,933 genome sequences from dogs, 214 from cats, 59 from humans, and 473 of unknown host origin (Table S1 includes the number of genome sequences per country). Multilocus sequence typing was performed separately on both the NA and global data sets using mlst (https://github.com/tseemann/mlst) and the PubMLST database (28). Core genome alignments were generated with Panaroo v.1.2.9 (27) using the MAFFT algorithm (29). Gaps were then eliminated using Gblocks v.0.91b (30, 31). Trees were built using IQ-TREE v.2.0.3 (32) and iTOL (33). hierBAPS population genomic clustering was conducted using the R package RhierBAPS (34), employing the same multiple sequence alignments used for phylogenetic reconstruction.

### Pan-genomics, plasmid identification, and Scoary

Panaroo v.1.2.9 (27) was used to demarcate the *S. pseudintermedius* pan-genome for both the NA and global sets of data and to identify core and accessory genes for downstream analysis. The interpretation data were compiled and coded into binary data for each reported antibiotic (0 = susceptible; 1 = resistant), and Scoary v.1.6.16 (15) was used to correlate resistance phenotypes with accessory gene presence/absence. Genes judged to be correlated with resistance needed to pass both empirical *P*-value and Bonferroni correction, at *P* < 0.01. The “collapse” flag was added to the command line of Scoary runs, since this collapses genes that are identically distributed in the sample set. This is the recommended approach from a statistical perspective because, in the opposite case, the program would test multiple identical null hypotheses penalizing the results in terms of multiple comparisons correction. We focused our attention on identifying putative novel resistance genes that were chromosomal, so we first identified known AMR loci, as well as genes carried on plasmids, because plasmid-borne genes may simply be correlated with resistance due to linkage with a known AMR locus. Scoary significant genes were compared with known AMR determinants using AMRFinderPlus (11). Plasmids were identified using PlasmidFinder (35). Gene clusters identified with Panaroo were designated as plasmid-borne if >10% of the members of a given cluster were located on the predicted plasmids. This procedure resulted in categorizing the Scoary significant genes into the following four groups, where “plasmid” in the following categorizations excludes chromosomally integrated plasmids: (i) known AMR locus carried on a plasmid; (ii) on a plasmid but not a known AMR gene; (iii) known AMR gene not on a plasmid; (iv) not a known AMR gene and not on a plasmid. SCCmec cassettes were typed with Staphopia (github.com/staphopia/staphopia-sccmec) (36).

## RESULTS

### Pan-genome, core-genome phylogeny, and population genetics

**Fig 1:**
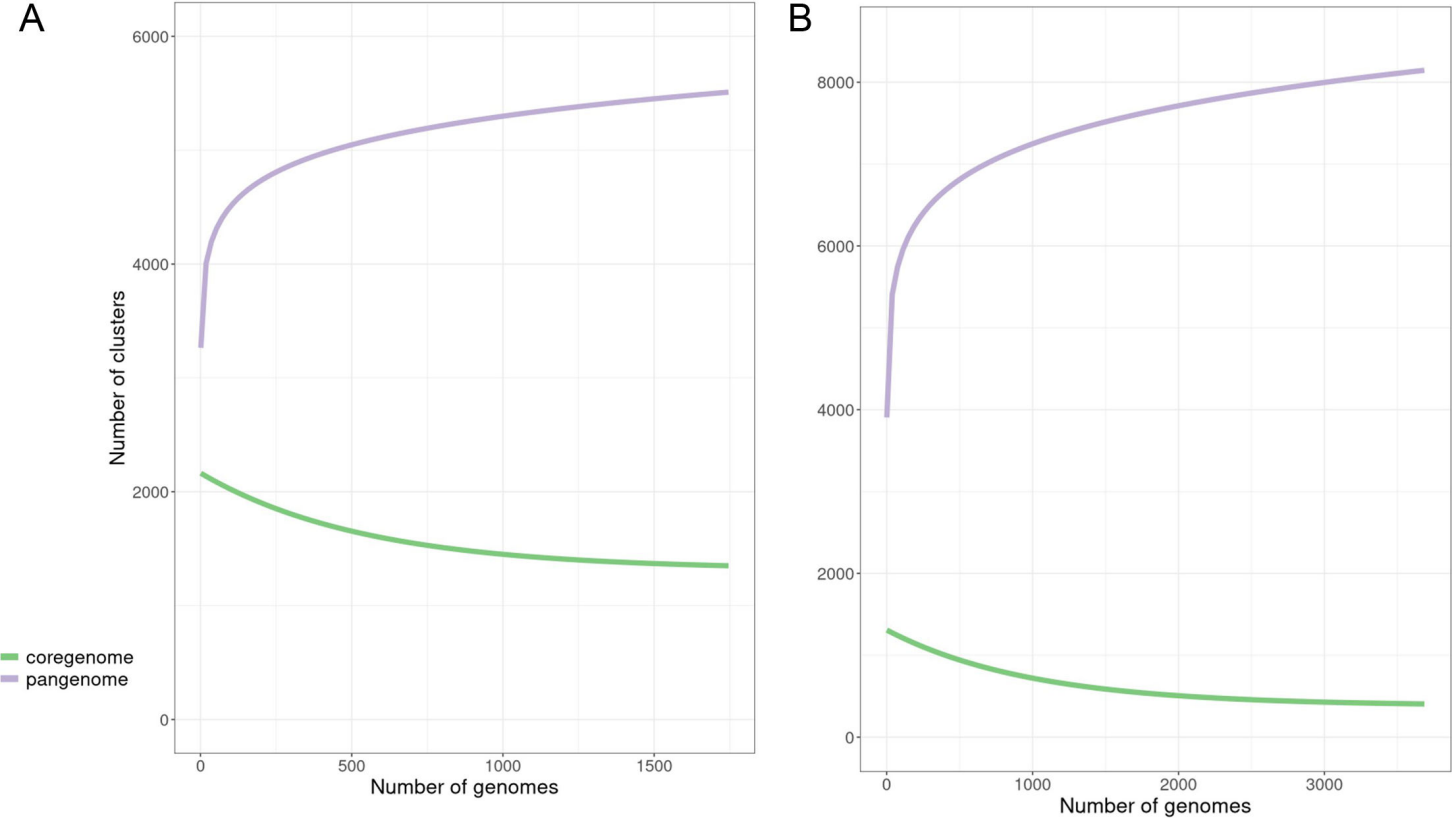
*Staphylococcus pseudintermedius* pan-genome curves. (**A**) FDA-USDA data set from North America. (**B**) Global data set inclusive of FDA-USDA.

**Fig 2:**
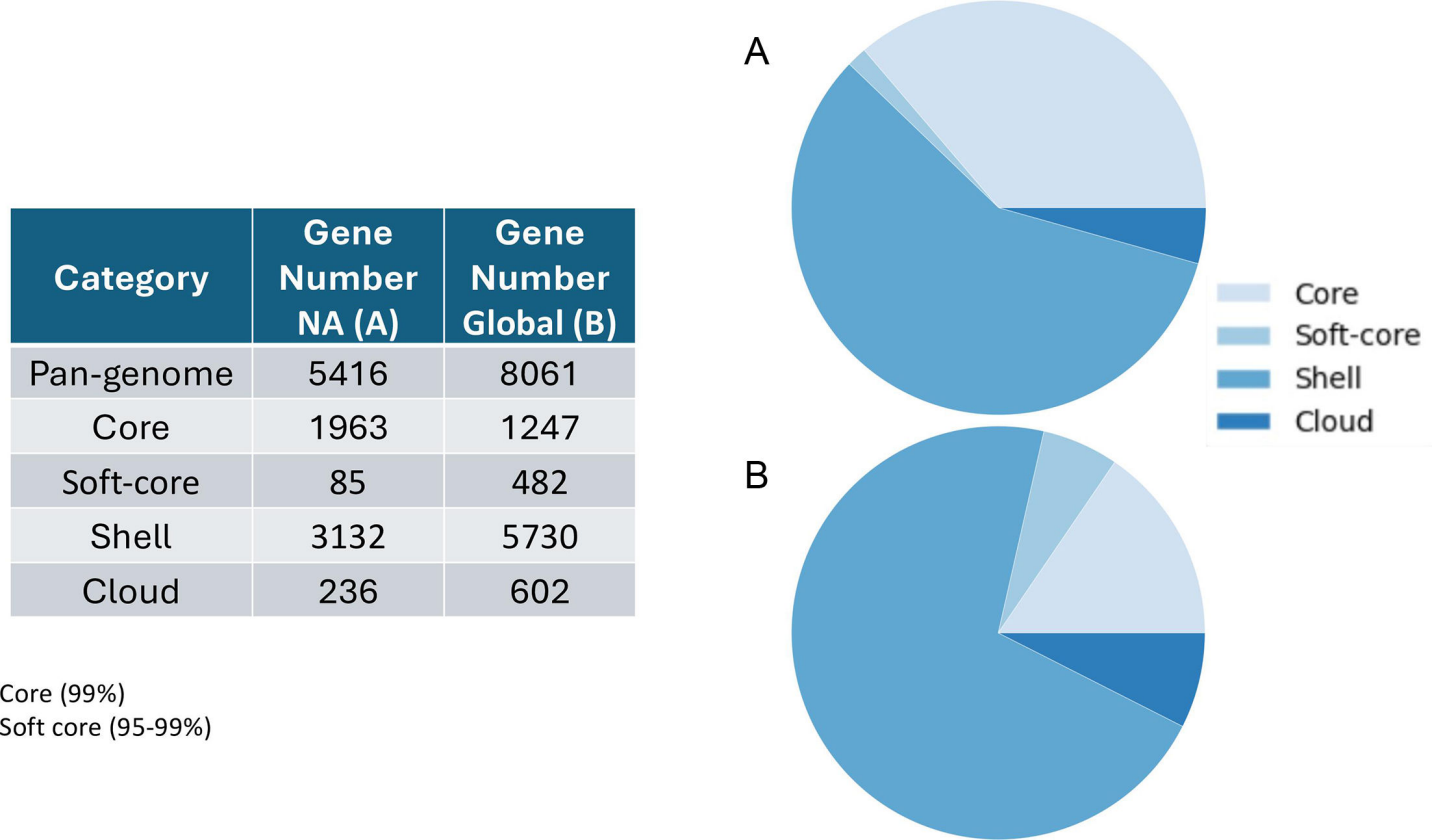
*Staphylococcus pseudintermedius* pan-genome statistics. (**A**) FDA-USDA North American data set. (**B**) Global data set inclusive of FDA-USDA.

**Fig 3:**
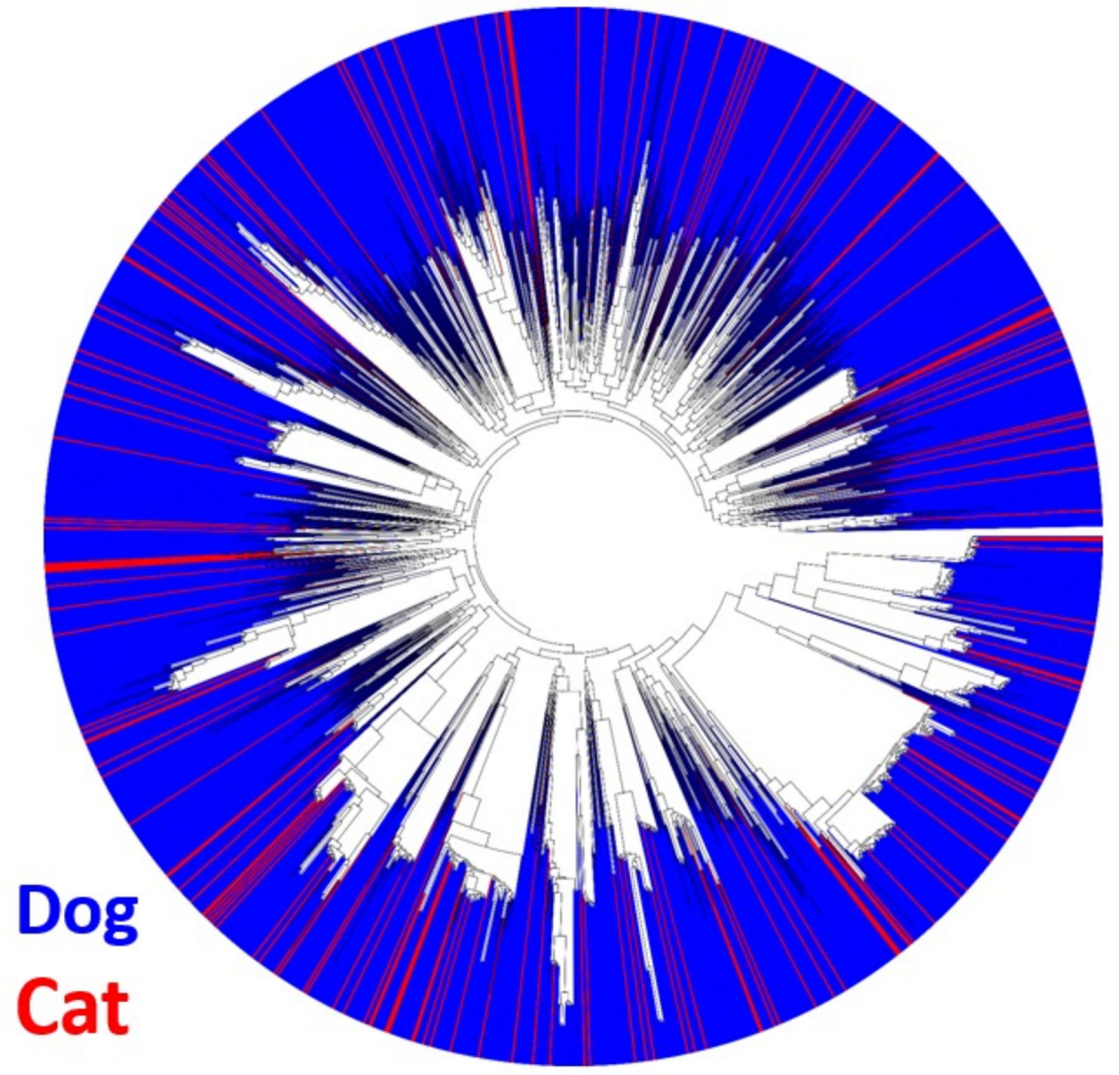
Core-genome phylogeny of *Staphylococcus pseudintermedius* isolates from the FDA-USDA North American data set, derived from cat and dog hosts inferred with IQ-TREE v.2.0.3, showing absence of host-specific lineages.

**Fig 4:**
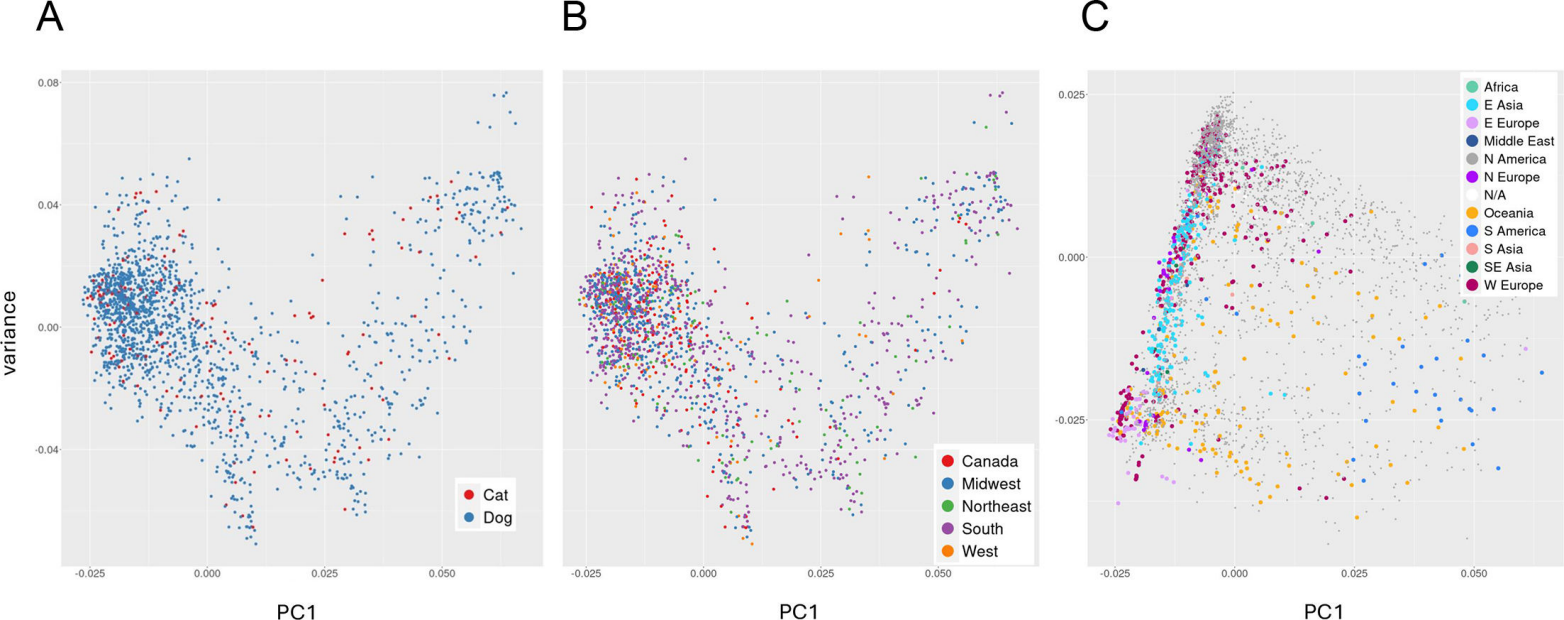
PCA of accessory gene content of *Staphylococcus pseudintermedius* isolates from different sample sets and perspectives. (**A**) Cat and dog FDA-USDA isolates, showing no clear distinction in gene content between hosts. (**B**) FDA-USDA isolates from different geographic regions showing no difference in gene content between areas. (**C**) Global comparative set showing different gene content between several regions, such as North America, E. Asia, and E. Europe. North America: USA, Canada; Oceania: New Zealand, Australia; W Europe: Spain, Netherlands, Germany, France, Italy, Ireland, United Kingdom, Switzerland, Belgium; E Asia: China, South Korea, Hong Kong, Japan; SE Asia: Thailand; E Europe: Slovenia, Hungary, Poland, Russia, Czech Republic; S Asia: Sri Lanka, India; N Europe: Norway, Sweden, Denmark; South America: Argentina, Puerto Rico, Brazil, Grenada; Africa: Botswana; Middle East: Israel, Turkey.

**Fig 5:**
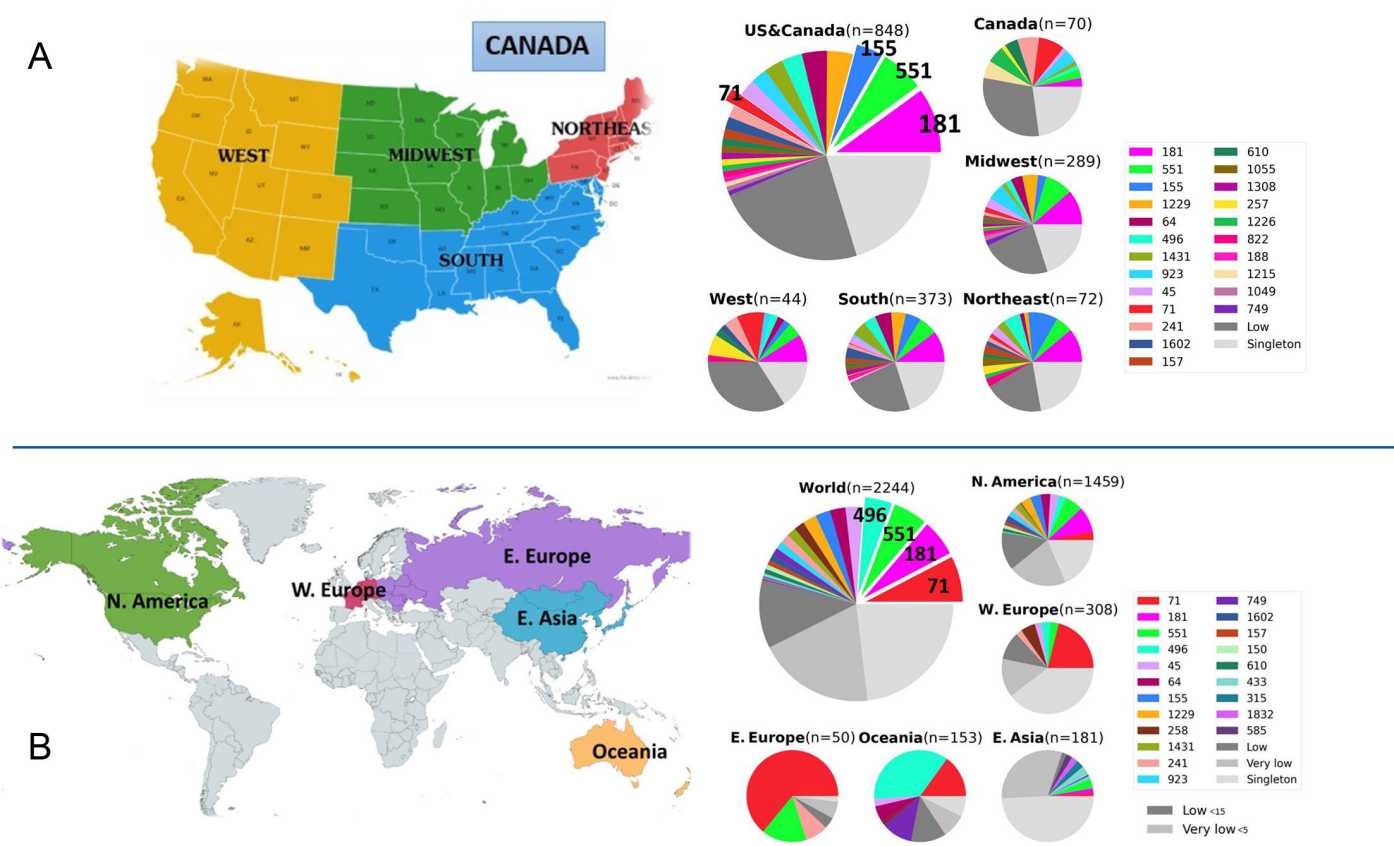
*Staphylococcus pseudintermedius* MLST results. (**A**) North American FDA-USDA set of isolates. (**B**) Global set of isolates. For both sample sets, the number of typable isolates was far less than the total number of isolates because of unique MLST allelic combinations not found on the pubMLST database. See Fig. 4 legend for countries included for each region. The map was plotted with the online tool https://www.mapchart.net/.

**Fig 6:**
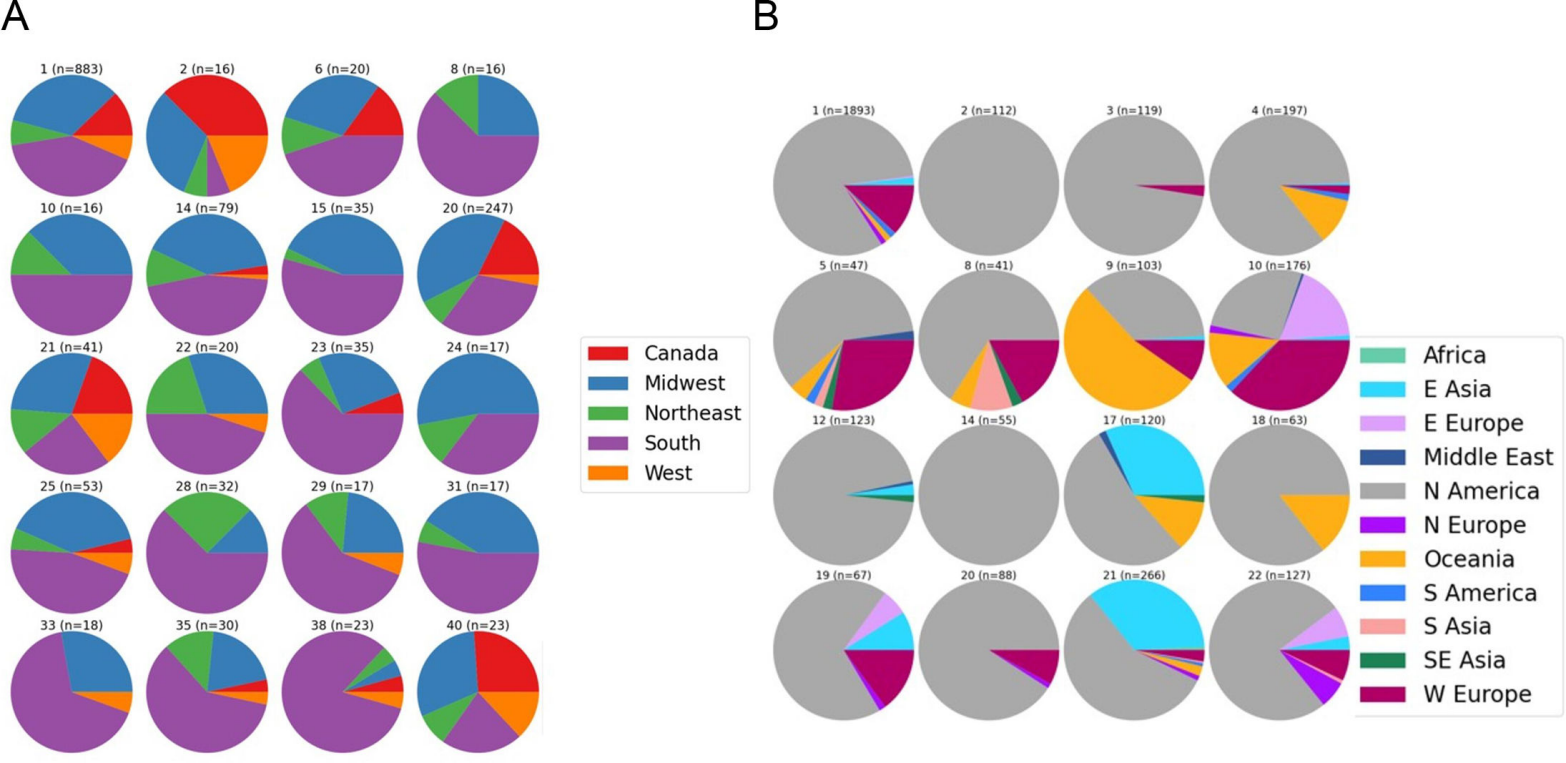
Bayesian analysis of population structure with hierarchical clustering (hierBAPS) of *Staphylococcus pseudintermedius* genome sequence data. (**A**) North American FDA-USDA isolates. (**B**) Global set of isolates. See the Fig. 4 legend for countries included for each region.

Pan-genomic components were determined separately for both the NA and global data sets. The data for both supported an open pan-genome with new genes being discovered with new genomes sequenced; the NA set peaked at 5,416 genes and had a core genome size of 1,963 (Fig. 1A). The complete pan-genomic statistics appear in Fig. 2A. The combined cat/dog core genome alignment for the NA set was 1,962,770 bp before curation (Gblocks) and 734,143 bp after curation. The core-genome phylogeny did not support single distinct monophyletic groups of cat and dog isolates, or alternatively several distinct lineages of cat and dog isolates (Fig. 3); instead, both host groups were entirely mixed. Principal component analysis (PCA) of the NA accessory gene content does not support a distinction in gene content between *S. pseudintermedius* isolated from the two different hosts (Fig. 4A) or from geographic areas (Fig. 4B). MLST profiles for the NA set indicated that ST181 was the most common of typable isolates, followed by ST551 (Fig. 5A). This pattern repeated itself in the different geographic regions, with the exception of Canada, where ST181 was less dominant. An abundance of low copy (≤5 and >1) and singleton sequence types (STs) was apparent in this NA set. There were also a large number of isolates without ST assignment on the pubMLST database. For the NA set, this included 898 isolates, and of those, 839 were unique allelic combinations. We found 40 distinct genetic groups that had an *N* > 10 for each cluster using RhierBAPS at hierarchical level 2 clustering. Within each of these clusters, there were samples from multiple distinct geographic regions, indicating that population structure is not strongly related to geography (Fig. 6A).

The open pan-genome curve of the global set of 3,685 isolates peaked at 8,061 accessory genes and 1,247 core genes (Fig. 1B and 2B). A core genome alignment of 3,685 global isolates had an alignment size of 1,774,479 bp before curation and 72,161 bp after Gblocks. A PCA of accessory gene content on the global set indicated that there were differences in gene content between several of the regions, including, for example, NA, E. Asia, and E. Europe (Fig. 4C). MLST profiles for the global set indicated that ST71 is the dominant, widely distributed, global sequence type and that ST181 is common in NA but relatively uncommon elsewhere (Fig. 5B). E. Asia has high ST diversity dominated by singletons and STs represented by very few isolates. In the global set, there were an additional 509 isolates without ST assignment on pubMLST and, of these, 474 unique allelic combinations. A total of 16 genetically distinct clusters were identified by hierBAPS at hierarchical cluster level 2, and with *N* > 20 for each cluster (Fig. 6B). Overall, there was evidence for global mixing of isolates, with some indication of genetically distinct clusters comprised of exclusively or indeed mostly NA isolates; however, our sample set is heavily biased toward NA isolates. The majority of E. Asian isolates were found in two separate clusters, suggesting some genetic distinction of samples from that region. Genetic distinction was also apparent between NA, E. Europe, and E. Asia isolates in the principal component analysis of accessory gene content (Fig. 4C).

### AMR

**Fig 7:**
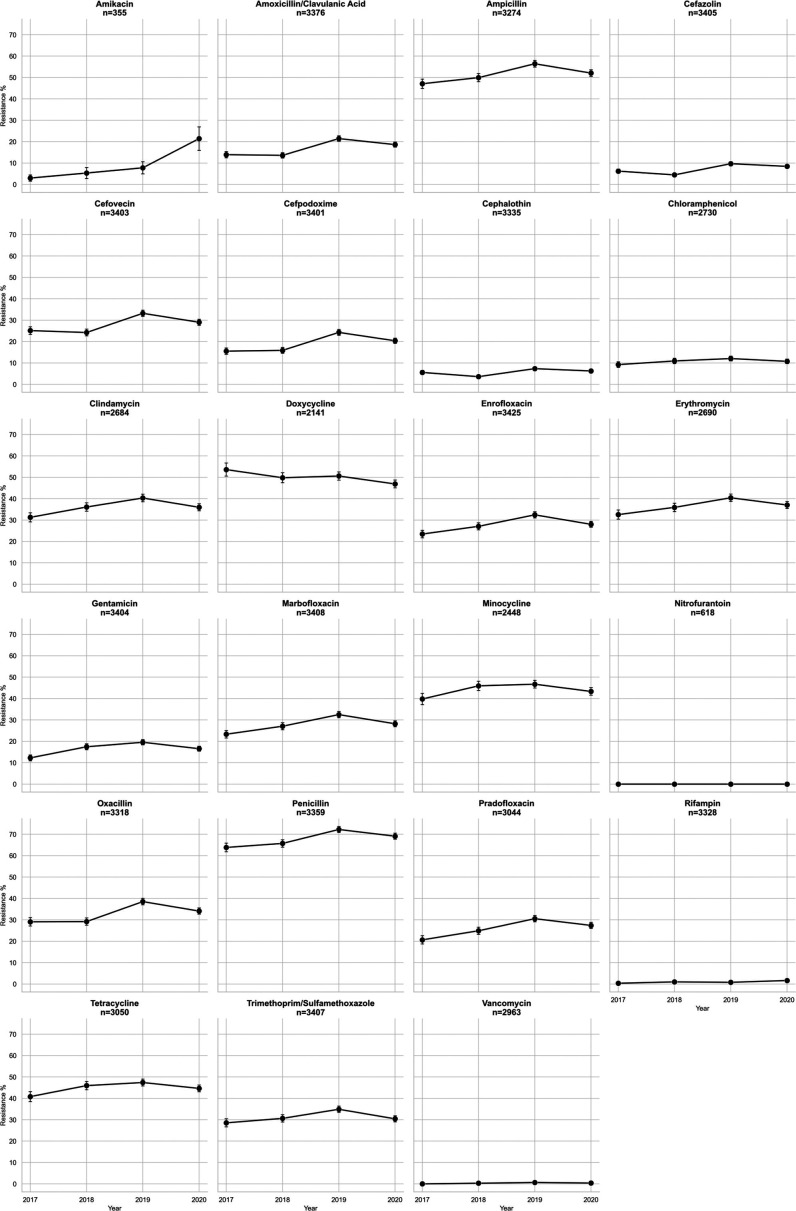
Trends in *Staphylococcus pseudintermedius* lab-tested antibiotic resistance for the period 2017–2020. The total number of samples included in each antibiotic trend analysis is indicated under each antibiotic, and standard error bars are marked on each point.

AMR laboratory-derived AST phenotype data were available only for the NA set of isolates. Several antibiotics showed little or no evidence for resistance over the 2017–2020 period, including rifampin, nitrofurantoin, and vancomycin; others showed an increase up to 2019, followed by a decline in 2020, with a notable exception being amikacin, which showed an increase in resistance between 2019 and 2020 (Fig. 7). A few had little evidence of change over the 4 years such as cefazolin and cephalothin; penicillin showed the highest level of resistance (Fig. 7). There were few notable differences in the proportion of isolates resistant across regions, except Canada, which showed lower levels of resistance for most antibiotics, except for the penicillins (Fig. S1).

**Fig 8:**
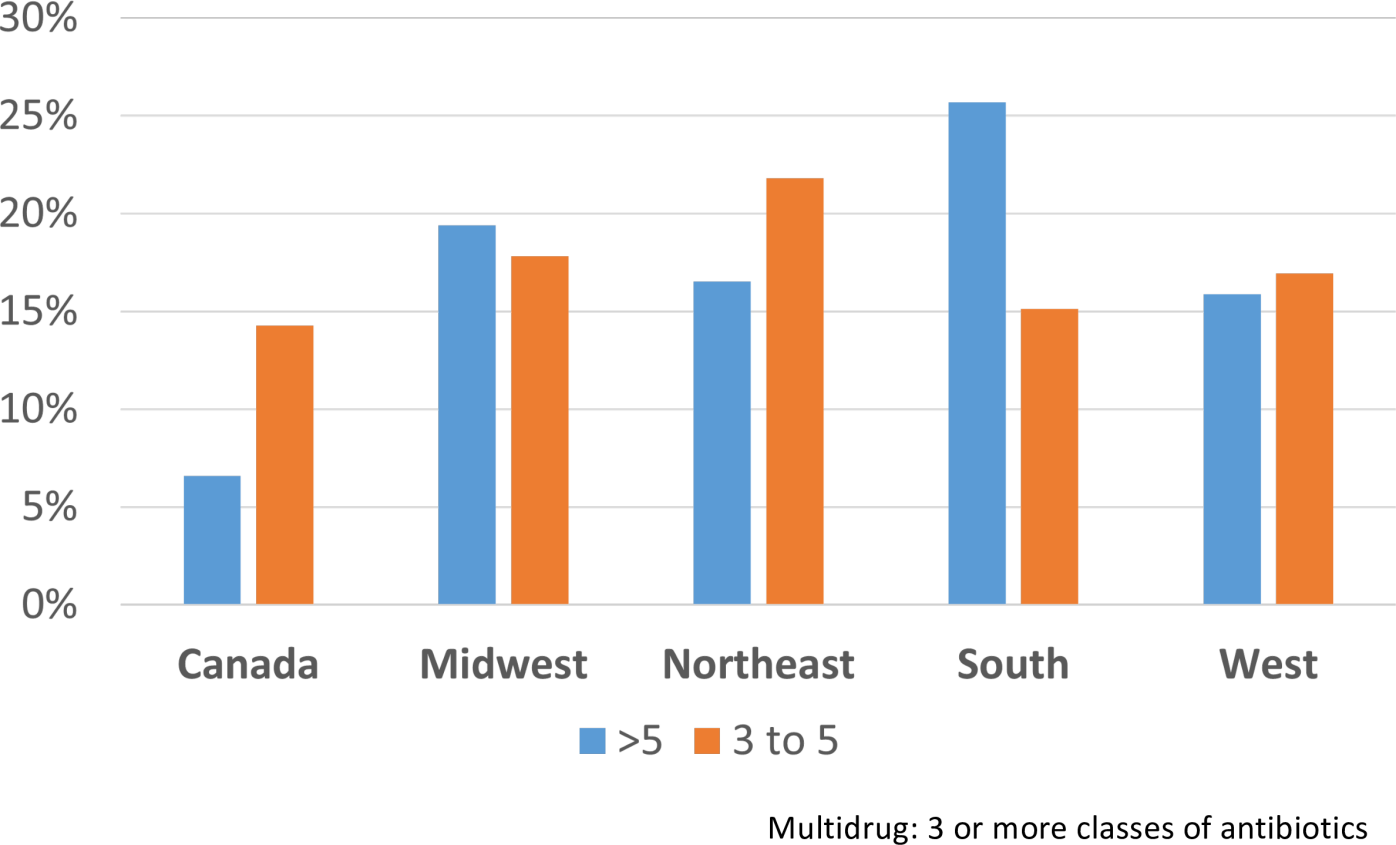
Percentage of lab-tested *Staphylococcus pseudintermedius* multidrug-resistant FDA-USDA isolates in different North American regions; 3 to 5 = resistant to three to five different classes of antibiotics; >5 = more than five classes of antibiotics. For category >5 antibiotics: chi-squared = 37.506, df = 4, *P*-value = 1.417e − 07 and multiple comparisons: South was significantly different from Canada (*P* = 0.00068) and from Midwest (*P* = 0.00049). Midwest was significantly different from Canada (*P* = 0.03709), and West was significantly different from South (*P* = 0.04095). All other comparisons were non-significant. For category 3–5 antibiotics: chi-squared = 7.0415, df = 4, *P*-value = 0.1337 (not significant).

**Fig 9:**
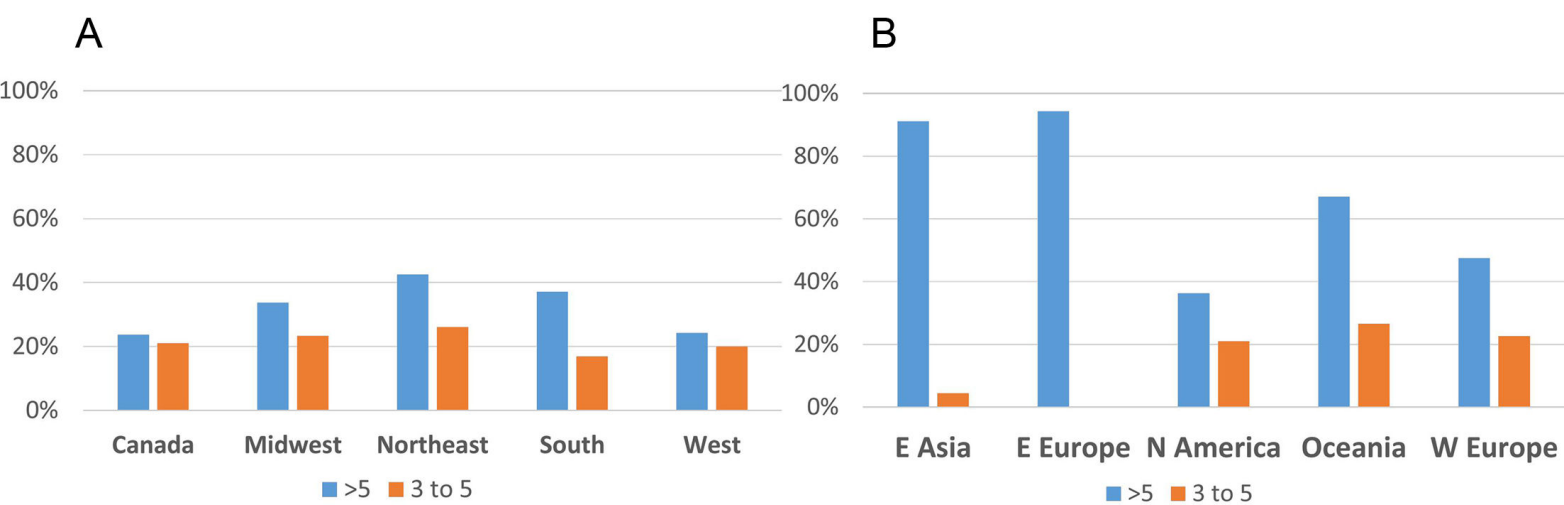
Percentage of multidrug resistance loci in *Staphylococcus pseudintermedius* genomes. (**A**) North American FDA-USDA isolates. (**B**) Global set of isolates. For NA >5 antibiotics: chi-squared = 20.615, df = 4, *P*-value = 0.0003775 and multiple comparisons: Canada was significantly different from Northeast (*P* = 0.0051) and from South (*P* = 0.0067). For NA 3–5 antibiotics: chi-squared = 11.494, df = 4, *P*-value = 0.02154 and multiple comparisons: South was significantly different from Midwest (*P* = 0.042). Statistics are not provided for the global set because many regions had few samples.

**Fig 10:**
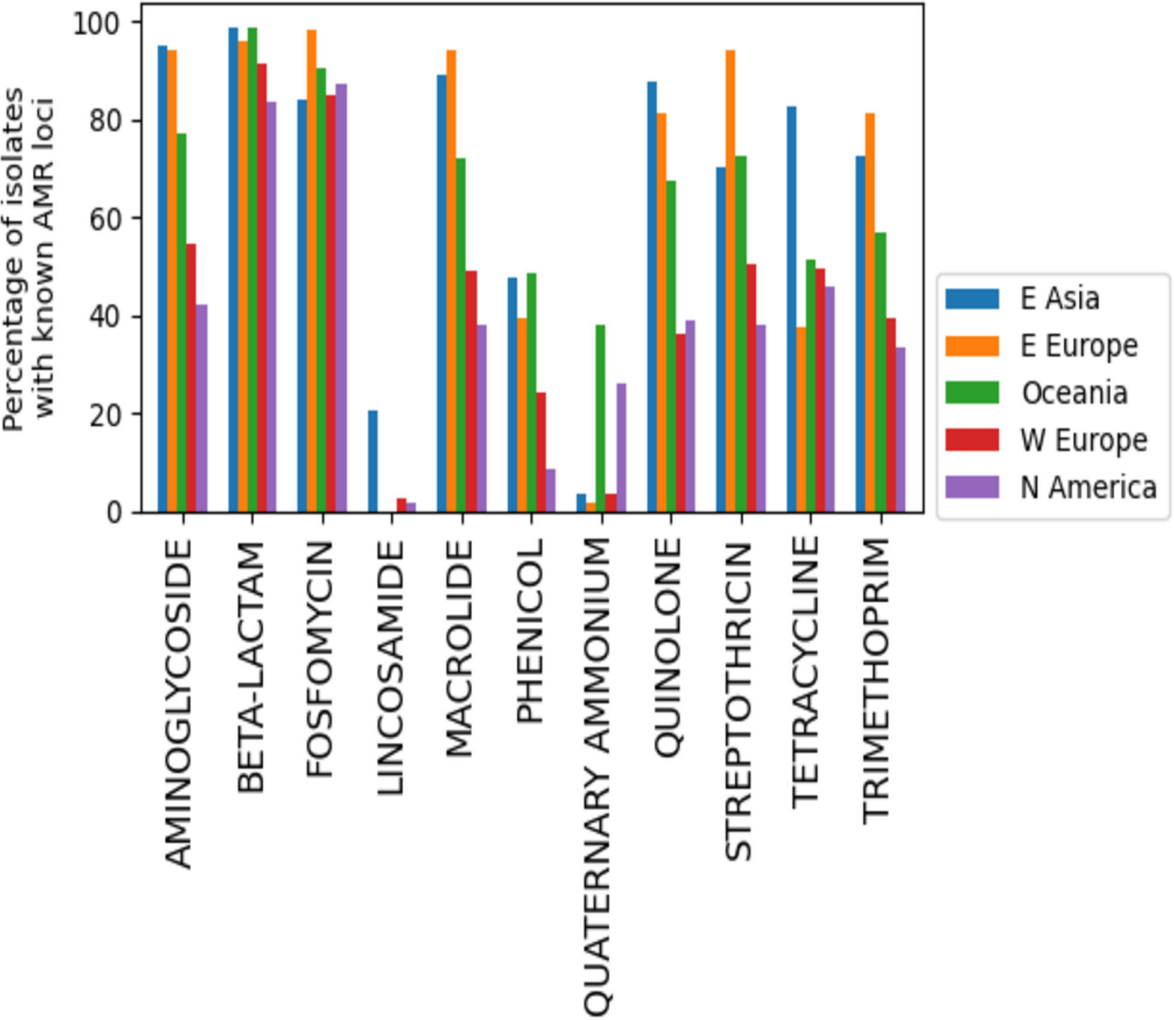
Percentage of *Staphylococcus pseudintermedius* genomes, from the global set of isolates, with known AMR loci to various antibiotic classes.

Multidrug resistance was evaluated at both >3 and >5 classes of antibiotics. In NA for laboratory-tested isolates, there were differences between areas, with the most notable difference apparent in the >5 category for the southern USA and 3–5 drug classes for Canada, although several other comparisons across regions were also different (Fig. 8). Based on known AMR genes, the percentage of isolates carrying multiple classes of AMR loci showed some minor differences in NA between regions (Fig. 9A), whereas in the global set, large differences were apparent between most regions, with very high proportions of isolates carrying >5 known AMR classes in E. Asia and E. Europe (Fig. 9B). When one considers the percentage of isolates in the global set carrying known AMR genes for different classes of antibiotics, there are notable differences between areas, including, for example, higher proportions in E. Asia, E. Europe, and Oceania for most antibiotics (Fig. 10). Aminoglycoside is one such class of antibiotics with higher proportions of resistance-determining loci compared to W. Europe and N. America. If one considers the contributions of different loci in the separation apparent between E. Asia and NA on the PC2 axis in the principal component analysis of global isolate accessory genomes (Fig. 4C), several of the top 10 loci (based on a ranking of significance) contributing to that separation are involved in conferring aminoglycoside resistance (Fig. S2). Several other mechanisms unique to NA were of note. The trimethoprim resistance gene *dfRS1* and quinolone resistance determinant *grlA*_D84H of DNA topoisomerase IV were unique to NA. Lincosamide resistance gene *lnu(G*) was unique to NA with the exception of one isolate from Kenya. Looking at more recent surveillance data not included in this study, extended spectrum beta-lactamase gene *blaTEM-116* was detected in three 2023 cases from Canada and has thus far not appeared in this species anywhere else in the world.

SCCmec is a genomic island that encodes methicillin resistance. SCCmec elements in *S. aureus* are classified into different types based on the combination of variants of mec and the cassette chromosome recombinase gene. This classification scheme was established for *S. aureus* and is the same one used for *S. pseudintermedius* (18, 37). Cassette type “mecA” was by far the most dominant type of SCCmec, with its greatest proportional abundance in S. Asia. Some differences in proportional abundance of SCCmec types between areas were apparent, with, for example, E. Europe and SE. Asia dominating in a couple different SCCmec types (Fig. S3A). Differences of SCCmec types between cat, dog, and human hosts were negligible (Fig. S3B).

### PAN-GWAS—Scoary

**Fig 11:**
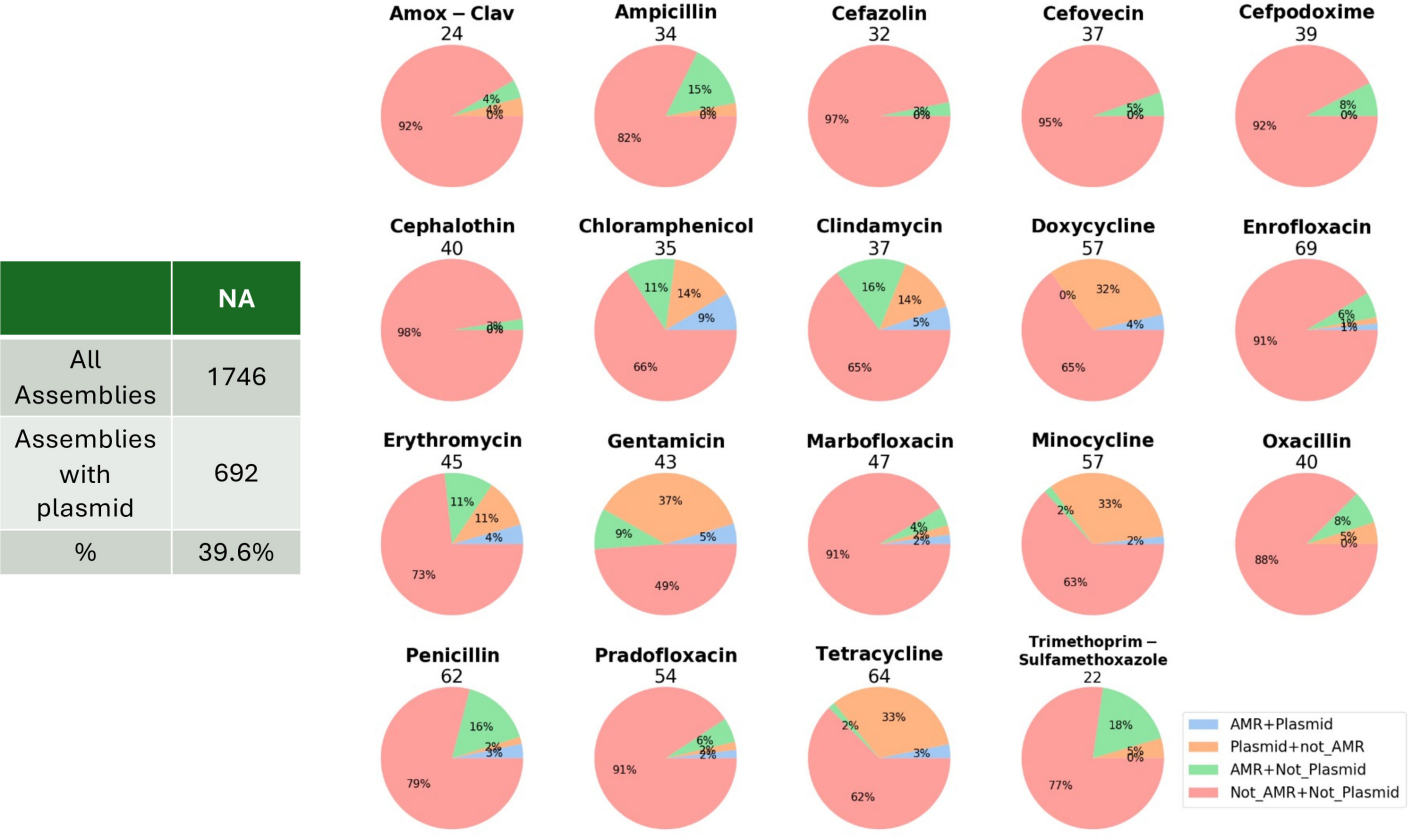
Pan-GWAS (Scoary) results for tested antibiotics. The numbers above the pie diagrams are the numbers of genes judged to be significantly correlated with resistance for that antibiotic, and their proportions are broken down into the four indicated components in these pie diagrams. AMR + plasmid = known AMR locus carried on a plasmid; plasmid + not AMR = gene carried on a plasmid that is not a known AMR locus; AMR + not plasmid = known AMR locus on the chromosome; not_AMR + not_plasmid = not a known AMR locus carried on the chromosome.

The number of genes significantly (*P* < 0.01 for empirical *P*-value and Bonferroni correction) correlated with resistance to the different antibiotics ranged from 22 to 69, with trimethoprim-sulfamethoxazole at the lower end of that range and enrofloxacin at the upper end. We divided the genes correlated with resistance in the Scoary analysis into four categories (Fig. 11). Most genes correlated with resistance are in the category “notAMR + notPlasmid,” which means they are likely on the chromosome and they are not identified as a known AMR mechanism. For some antibiotics, there are very few known AMR genes identified, e.g., some of the cephalosporins, as penicillin-binding protein mutations are often responsible and not identified by AMRFinderPlus. On average, based on the presence and absence of Scoary significant genes, ~89% of phenotypically resistant isolates carried a known AMR locus (Table S2). Several of the antibiotics have a relatively high proportion of the Scoary significant genes identified as on plasmids, but they are not known AMR mechanisms, suggesting these are likely plasmid cargo genes, genes carried with the plasmid, along with a known AMR mechanism. We exclude these from further consideration as possible novel mechanisms since there is no bioinformatic means of separating them from their linkage with the assumed known AMR mechanism carried on the plasmid. PlasmidFinder identified approximately 40% of 1,746 NA genomes as carrying at least one plasmid, and about 46% in the global set of data. Most genomes were assessed as carrying only a single plasmid. Hypothetical genes, those which were annotated as “hypothetical,” comprised the largest group of genes correlated with resistance across all antibiotics, ranging from 44.44 to 80% of the genes for individual antibiotics (Table S3). Not surprisingly, the top 10 genes correlated with resistance (ranking based on level of significance) for each antibiotic included at least a few hypotheticals and, frequently, the majority. Often, these were likely plasmid cargo genes, for example, in the case of tetracyclines (tetracycline, doxycycline, minocycline) where the resistance mechanism carried on the plasmid was *tetM*. On other occasions, the same hypothetical, which was judged not to be on a plasmid and therefore likely chromosomal, appeared across several antibiotics in the same class, suggesting something of note for possible further experimental evaluation. An example would be hypothetical protein Panaroo group_957, which was the top-ranked Scoary significant gene for fluoroquinolones. Examples of functionally annotated genes that appeared in the top 10 most abundant Scoary significant loci (based solely on gene presence/absence) across antibiotics tested, and which were not known resistant genes, included glycerophosphodiester phosphodiesterase (ugpQ_2), ubiquinone biosynthesis O-methyltransferase (COQ3), and *Hin* DNA invertase. As expected, *mecA* was identified to be associated with all beta-lactams in this study. A complete list of genes significantly correlated with resistance is available in Table S3.

## DISCUSSION

### Population genetics and genomics

This manuscript presents an analysis of the largest *S. pseudintermedius* genomic data set yet assembled, concomitant with AMR data of 23 antibiotics (19 with sufficient data for pan-GWAS) from over 1,700 dog isolates from NA, and a pan-GWAS analysis of these different antibiotics across these genomes. Along with the dog genomes from NA, 176 cats were also completed. Although this number was far less than the canine sample set, it was sufficient to indicate that there was no separate adaptive lineage of cats relative to dogs. This was apparent at both the level of a core-genome phylogeny of dog and cat isolates as well as PCA of their gene content. Similar findings have recently been reported involving human isolates, where genome architecture was indistinguishable between dogs and human hosts (1). These authors did, however, find some genetic distinction in niche-specific (diagnostic versus colonizing) genes involved in defense. We suggest that future molecular evolution analyses could possibly identify site-specific genetic features of molecular adaptation between cats and dogs in immune evasion genes shared between each host.

Our MLST analysis on this large set of *S. pseudintermedius* genomes provided evidence for clonal expansion of certain dominant sequence types in NA that had both similarities and differences to that on a global scale. ST181 was the dominant sequence type throughout the USA but not so in Canada where ST71 was dominant. ST181 is an MRSP multidrug-resistant clone that appears to have replaced other sequence types such as ST71 (also MRSP) as the dominant clone in the USA over the course of the last decade (38). In a recent study, Bruce et al. (8) found that of 170 *S*. *pseudintermedius* isolates, ST71 was absent from New England, although present in a few other states. Nevertheless, over the 2017–2020 period of our present collection, ST71 remains a prevalent clone in the western USA and in Canada. Two other STs well represented in the NA set are STs 551 and 155. ST551 is an MRSP clone that has recently been reported as a commensal in dog skin (39), suggesting this highly drug-resistant clone could be an important one to watch for in zoonotic infections. ST155 is an MRSP clone that has been reported in human infections, as have ST45, ST241, ST1337, ST1412, ST673, ST686, ST181, ST158, and ST233 (6), several of which were identified in our NA set (Fig. 5A).

In the global set of data, ST71 was the dominant clone, with ST181 only common in NA. ST71 is reported as the most prevalent clone in other analyses of European *S. pseudintermedius*, although its dominance is suggested to be declining over the course of the last decade (40–43), and gradually being replaced by ST258 (41, 44), at least in western Europe. ST258 is reported to be more susceptible to licensed veterinary antimicrobials, including fluoroquinolones (40, 45). We found ST258 to be rare in NA. Our analysis divided the European data into western and eastern regions, and although the sample size for E. Europe was much less, there were several differences apparent between the two regions. E. Europe had a much higher proportion of the isolates represented by ST71 and ST551, and much lower overall ST diversity than Western Europe. The last two regions in our global ST categorization—Oceania and E. Asia—had their own unique ST profiles. Oceania showed a distinct dominance of ST496, which was rare elsewhere in the world. Earlier reports on *S. pseudintermedius* from Australia reported the first occurrence of this multidrug-resistant clone ST496 and suggested that it may have evolved on that continent, possibly in New South Wales (37). Our analysis suggests that it is no longer exclusively found in Australia—we detected it in NA and Western Europe, albeit in low numbers. At the time of Worthing et al.’s (37) study, ST71 was the dominant ST, but our results suggest that, like other parts of the world, ST71 dominance in Australia appears to be waning and, in this case, being replaced by ST496. E. Asia differed from other regions of our global sample set in having the highest ST diversity, with the vast majority of the sampled STs (*N* = 181) being represented by less than five isolates. Although accurate comparisons across studies can be hampered because of large differences in sample sizes, it nonetheless does appear that clonal structure of *S. pseudintermedius* is in flux, possibly related to lateral acquisition of SCCmec cassettes or other resistance-determining loci that then allow certain clones to gain dominance.

The hierBAPS population genomics analyses provide a different perspective on genetic differences across regions, this time based on curated core genome alignments, rather than just fragments of seven housekeeping genes. Our study provides, for the first time, adequate sample numbers across NA to provide any attempt at evaluating whether there might be genetically distinct pockets of *S. pseudintermedius* in the USA and Canada. In NA, the clear result was that there is no support for population genetic geographic clustering; the genetically distinct clusters identified by hierBAPS comprised multi-geographic regions. In the global data set, the hierBAPS population genomics did identify several genetic clusters that were either exclusively NA or very nearly; the remaining genetic clusters were mixed in regional composition. The distinction apparent in NA compared to much of the rest of the world is undoubtedly impacted by the much larger sample size for NA, which could conceivably result in additional genetically distinct clusters. However, some of these NA clusters are nonetheless substantial in the numbers represented, often over 100 isolates.

When considering the accessory genome content, differences were observed between geographic areas in the global data set, including a tendency toward unique content in regions such as E. Europe, NA, and E. Asia, and to a lesser extent, Oceania and South America. Some of this difference in gene content is at least partly a consequence of differences in the content of AMR loci, which our results support when this is broken down into genes conferring resistance to different classes of antibiotics across regions (Fig. 11). These differences in turn may reflect differences in antibiotic prescribing and usage habits across different countries. Recent analysis of this topic for human pathogens, including *S. aureus*, has developed indices that consider levels of resistance, as well as their association with antibiotic consumption (46). Evaluations with such indices in mind have concluded that higher levels of resistance in E. Asia, as well as in some E. European countries, may be a consequence of inappropriate antibiotic consumption (46), including the use of antibiotics to treat conditions not related to a bacterial infection, the use of the wrong antibiotic, the wrong dosage, or the wrong duration. Similar detailed global comparisons of AMR in companion animal medicine are not available due to various factors, including limitations on the number of dilution steps available on commercial diagnostic panels that preclude obtaining accurate MIC or data. The majority of drugs in our analysis contain four or fewer dilutions and are therefore subject to the same limitations. However, if one assumes that poor antibiotic stewardship in human medicine of some countries would also be true in animal medicine of those same countries, then this might explain the higher levels of known AMR mechanisms in *S. pseudintermedius* in E. Asia and E. Europe. AMR comparisons, especially those that may contain unbiasedly sampled sequences (sequences targeted specifically because of resistance or some other highlighted phenotype), may then influence downstream analyses, and thus should be considered as a possible limitation for this study. Furthermore, *S. aureus* does infect dogs and cats (47, 48), and human to dog transmission of *S. aureus* has also been documented (49, 50). Both E. Europe and E. Asia are regions with high incidence of methicillin-resistant *S. aureus* (MRSA) (51–53). Thus, human to dog transmission of *S. aureus* may occur with subsequent lateral gene transfer of AMR loci between *S. aureus* and *S. pseudintermedius* as a possible outcome, particularly since they are closely related organisms and share many of the same SCCmec cassettes and plasmids.

### Pan-GWAS

Our pan-GWAS analysis performed with Scoary identified a number of loci correlated with resistance that were judged to be chromosomal and were not known AMR loci. The number of genes significantly correlated with resistance to each of the tested antibiotics ranged from 22 to 69 (Fig. 11), and for each antibiotic, hypothetical proteins dominated the list (Table S3). In several instances, the same chromosomal hypothetical protein was found to be correlated with resistance to multiple antibiotics, suggesting reason for further laboratory experimental evaluation regarding these loci. Similarly, numerous chromosomal annotated genes that were not known AMR mechanisms were correlated with resistance to multiple classes of antibiotics. Examples of this included the CRISPR-associated endoribonuclease proteins Cas1, Cas2, Cas6, Csm3, and Csm4. CRISPR-Cas systems provide the bacteria adaptive immune protection against foreign mobile genetic elements. Various articles have focused on the CRISPR-Cas system as a means of limiting AMR development by restricting the acquisition of mobile elements, including those which carry resistance cassettes, and the possible use of this system to treat AMR pathogens (54–56). Our results, on the other hand, suggest a role for CRISPR-Cas systems in promoting antibiotic resistance. An earlier study on *Francisella novicida* provided experimental evidence that, in this species, the CRISPR-Cas system was promoting AMR and indicated that this involved enhancing envelope integrity through the regulation of a bacterial lipoprotein (57). We are not aware of the CRISPR-Cas system reported as a potential AMR mechanism in *Staphylococcus,* and we suggest this deserves consideration in not only *S. pseudintermedius* but possibly also *S. aureus*.

Other Scoary significant genes were not putative multidrug resistance mechanisms but instead were correlated with a single class of antibiotics. Several genes from the sbo-alb operon, responsible for the biosynthesis of subtilosin A, a bacteriocin produced by *Bacillus subtilis* (58), were correlated with macrolide resistance in our analysis. The genes from this operon correlated with macrolide resistance were *sboA*, *albA*, *albB*, and *albC. sboA* is responsible for subtilosin A production. AlbA catalyzes thioether bond formation in subtilosin A (59). AlbB is a small hydrophobic peptide involved in subtilosin immunity (60). AlbC is an ABC transporter thought to be involved in export of subtilosin A (60). SboA has been shown to have antimicrobial activity against a wide range of gram-positive and gram-negative bacteria (61). The protein products of *albB*, *albC*, and *albD* function in subtilosin immunity (60) which is necessary because a bacteriocin-producing bacterium needs to be immune to the bacteriocin it produces. We found the sbo-alb operon to be widely distributed among *S. pseudintermedius* isolates from our cats (19) and dogs (546; see Fig. S4 for the diagram of the *S. pseudintermedius* operon). An intact operon in *S. pseudintermedius* appears to consist of *sboA, albA, albB, albC, albD*, and *albE*; however, we do not have transcriptome data to verify its functional transcription. A complete operon in *Bacillus subtilis* consists of these same loci, with the addition of a small *sboX* locus between *sboA* and *albA* (*sboX* in *B. subtilis* is a very short peptide with an overlapping reading frame with *sboA*, and based on our homology comparisons, it is not clear whether *S. pseudintermedius* carries this locus) as well as *albF* and *albG* (58). BLAST searches of NCBI identified homologs from *Staphylococcus felis* (within which it appeared to be relatively common), *Staphylococcus epidermidis* (again relatively common), and a few representative isolates of several other species of *Staphylococcus* including *capitis, agnetis, caprae, delphini*, but completely lacking from *S. aureus*. A recent study has shown that topical applications of bacteriocins from *B. subtilis* can result in decolonization of skin infections by *S. aureus* (62). Subtilosin A can inhibit biofilm formation in clinical *S. aureus* strains (63) and *Bacillus subtilis* lipopeptides derived from cell-free extract disrupt quorum sensing and biofilm assembly in *S. aur*eus (64). Thus, the absence of this locus in *S. aureus* is likely because the species does not have the ability to withstand the production of this bacteriocin. *S. epidermidis,* on the other hand, does appear to carry this operon and is a closely related species to *S. aureus* (65). Given the distribution of this operon across numerous *Staphylococcus* species and, in particular, those closely related to *S. aureus*, such as the Epidermidis species group (65), the most parsimonious hypothesis is that this locus was present in at least the ancestor of the Epidermidis/Aureus and Hyicus/Intermedius cluster groups, and possibly deeper in the radiation of *Stapylococcus* species, and was then lost in *S. aureus*. Subtilosin A is an example of a radical SAM enzyme (S-adenosylmethionine) that can catalyze sulfur-to-alpha carbon thioether cross-linked peptides (referred to as sactipeptides), which is a sub-class of ribosomally synthesized and post-translationally modified (in this case by AlbA) peptides (RiPPs) (66). Sactipeptides are RiPPs that have various biological properties such as antibacterial, spermicidal, and hemolytic. Macrolide resistance is often conferred by modification, most commonly methylation, of the ribosomal target. Some radical SAM proteins catalyze unusual methylations including of rRNA ([67]; although not subtilosin to our knowledge). Possibly, the macrolide resistance we see in these *sboA*-carrying isolates is related to an rRNA modification inherited with the *sboA* operon that facilitates the production of the subtilosin ribosomal peptide, as well as macrolide resistance. Whatever the possible link between macrolide resistance and subtilosin A in *S. pseudintermedius,* the growing interest in using this bacteriocin as an agent to fight *S. au*r*eus* infections, particularly those resistant to multiple antibiotics, warrants further investigation in *S. pseudintermedius*, a closely related species to *S. au*reus, possibly making the *S. pseudintermedius* peptide a useful agent in such endeavors.

Numerous other putative resistance loci of note were identified in our pan-GWAS analysis. Several different recombinases were correlated with resistance*—xerC, hin*, and *pinR*. Certain antibiotics result in double-strand DNA breaks in bacterial chromosomes, which need to be repaired (the SOS response). *xerC* has recently been established as one of the genes in the *S. aureus* SOS response, and deletion of this gene increased the susceptibility of *S. aureus* to various classes of antibiotics (68). DNA invertase *hin* is a member of the hin family of site-specific recombinases, and in our analysis was correlated with resistance to 11 different antibiotics. *pinR* is a serine recombinase that facilitates the process of genetic material exchange between organisms through DNA recombination (69). Antibiotics, such as quinolones, aminoglycosides, and β-lactams, are known to induce oxidative stress and redox alterations that contribute to bacteria cellular damage and death (70). Several of the genes from the myo-inositol (*iol*) operon were correlated with resistance in our analysis—this operon is involved in protecting the bacteria from oxidative stress (71). There were a few examples of genes that are known resistance mechanisms for a certain class of antibiotics but were correlated in our analysis with resistance for different classes of antibiotics. For these specific antibiotics, we consider them as not a known resistance mechanism (i.e. not listed as such on AMRFinderPlus). An example of this is streptothricin acetyltransferase A, correlated in our analysis with resistance to six antibiotics from different classes. Another example is two aminoglycoside resistance mechanisms—aminoglycoside 3´-phosphotransferase, *aphA,* and aminoglycoside 6-adenylyltransferase, *aadK*, correlated with multiple classes of non-aminoglycoside antibiotics in our Scoary results.

Methicillin-resistant *S. pseudintermedius* and *S. aureus* are a major health concern for a number of reasons, including, for example, (i) these strains are resistant to methicillin (oxacillin), an antibiotic that is active against many penicillinase-producing strains of *Staphylococcus* that are resistant to other penicillins; (ii) they generally also carry resistance loci to a range of other classes of antibiotics; (iii) they are widely distributed, including increasing prevalence in hospitals; and (iv) the principal molecular agent of resistance—the *mecA* gene—is carried on a lateral gene transmissible (LGT) SCCmec cassette. Methicillin is no longer commercially available in the USA, and oxacillin has replaced it because it maintains better activity during storage and is better at detecting heteroresistant strains. The acronym MRSA is nonetheless still used to describe these isolates because of the historic role of methicillin. In our NA collection of *S. pseudintermedius*, *mecA* was found in 89% of the isolates judged to be oxacillin resistant and only 3.3% of the oxacillin-sensitive isolates carried *mecA*. Our results suggest, and it does remain an open question as to whether, there might be additional loci (72–74) that could be contributing to oxacillin resistance. In our analysis, the vast majority of Scoary significant genes for oxacillin were hypotheticals. The few annotated putative chromosomal genes (i.e., other than *mecA*) that were correlated with resistance included glycerophosphodiester phosphodiesterase cytoplasmic (*ugpQ*), hydroxymethylglutaryl-CoA synthase (*mvaS*), maltose O-acetyltransferase (*maa*), HTH-type transcriptional repressor (*glcR*), Na(+) Li(+) K(+)/H(+) antiporter (*mdrP*), DNA invertase (*hin*), HTH-type transcriptional regulator (*pchR*), beta-lactamase (*blaZ*), and protein adenylyltransferase (*soFic*). This latter gene (*soFic*) is a member of the Fic family of proteins which can induce the SOS response (75), for controlling DNA damage, one consequence of antibiotics, to which bacteria need to respond. It remains plausible that other contributors and not just the *mecA* gene are playing a role in oxacillin resistance; perhaps some of the loci on our oxacillin resistance list (Table S3) warrant some laboratory scrutiny in this regard. Similar to oxacillin resistance, other antibiotics in our analysis had approximately 10% of tested isolates that were resistant but did not carry a known AMR gene, further suggesting the possibility of unknown AMR loci for antibiotics more broadly (see Table S2).

There are a wide range of interesting potential antimicrobial loci identified in our analysis, but these are all correlations and would need experimental evaluation for cause-effect relationships to AMR. Furthermore, genotypic correlations to phenotypes may change as the AST interpretations are updated or if infections are transmitted to other hosts or body sites. Our purpose here was to provide a short list of candidate genes for the community of interested scientists to evaluate as they see fit. It is our hope this can be useful and could lend insight on control of AMR in not only *S. pseudintermedius* but possibly also in *S. aureus*.

## Data Availability

This study utilized genome sequences that were publicly available in NCBI. Genome sequences, metadata, and susceptibility phenotypes for the dog and cat isolates generated by the FDA and USDA monitoring programs are available in NCBI BioProjects PRJNA314609 and PRJNA510565. An aggregate version of the phenotypic and genotypic data for the dog isolates can be viewed via an interactive Tableau report at the following link: https://www.fda.gov/animal-veterinary/national-antimicrobial-resistance-monitoring-system/2021-animal-pathogen-amr-data.

## SUPPLEMENTAL MATERIAL

Fig. S1 aem.00010-25-s0001.pdf Percentage of lab tested isolates resistant to various antibiotics across the geographic regions of North America.

Fig. S2 aem.00010-25-s0002.pdf Genes that were the greatest contributors to separation of the PC2 axis in the principal component analysis of accessory gene content.

Fig. S3 aem.00010-25-s0003.pdf Distribution of SCCmec types.

Fig. S4 aem.00010-25-s0004.pdf sbo-alb operon.

Supplemental legends aem.00010-25-s0005.docx Legends for all supplemental material.

Table S1 aem.00010-25-s0006.xlsx Number of isolates represented for each country used in this study.

Table S2 aem.00010-25-s0007.xlsx Percentages of phenotypically resistant isolates containing a known AMR gene that is also Scoary significant by total number of phenotypically resistant isolates for each antibiotic tested.

Table S3 aem.00010-25-s0008.xlsx Scoary significant genes for each antibiotic tested.

ASM does not own the copyrights to Supplemental Material that may be linked to, or accessed through, an article. The authors have granted ASM a non-exclusive, world-wide license to publish the Supplemental Material files. Please contact the corresponding author directly for reuse.
