# Supplementary figures and images for: Population and pan-genomic analyses of Staphylococcus pseudintermedius identify geographic distinctions in accessory gene content and novel loci associated with AMR
Source: Appl Environ Microbiol. 2025 Apr 24;91(5):e00010-25. doi: 10.1128/aem.00010-25 (PMC12094015; doi:10.1128/aem.00010-25)

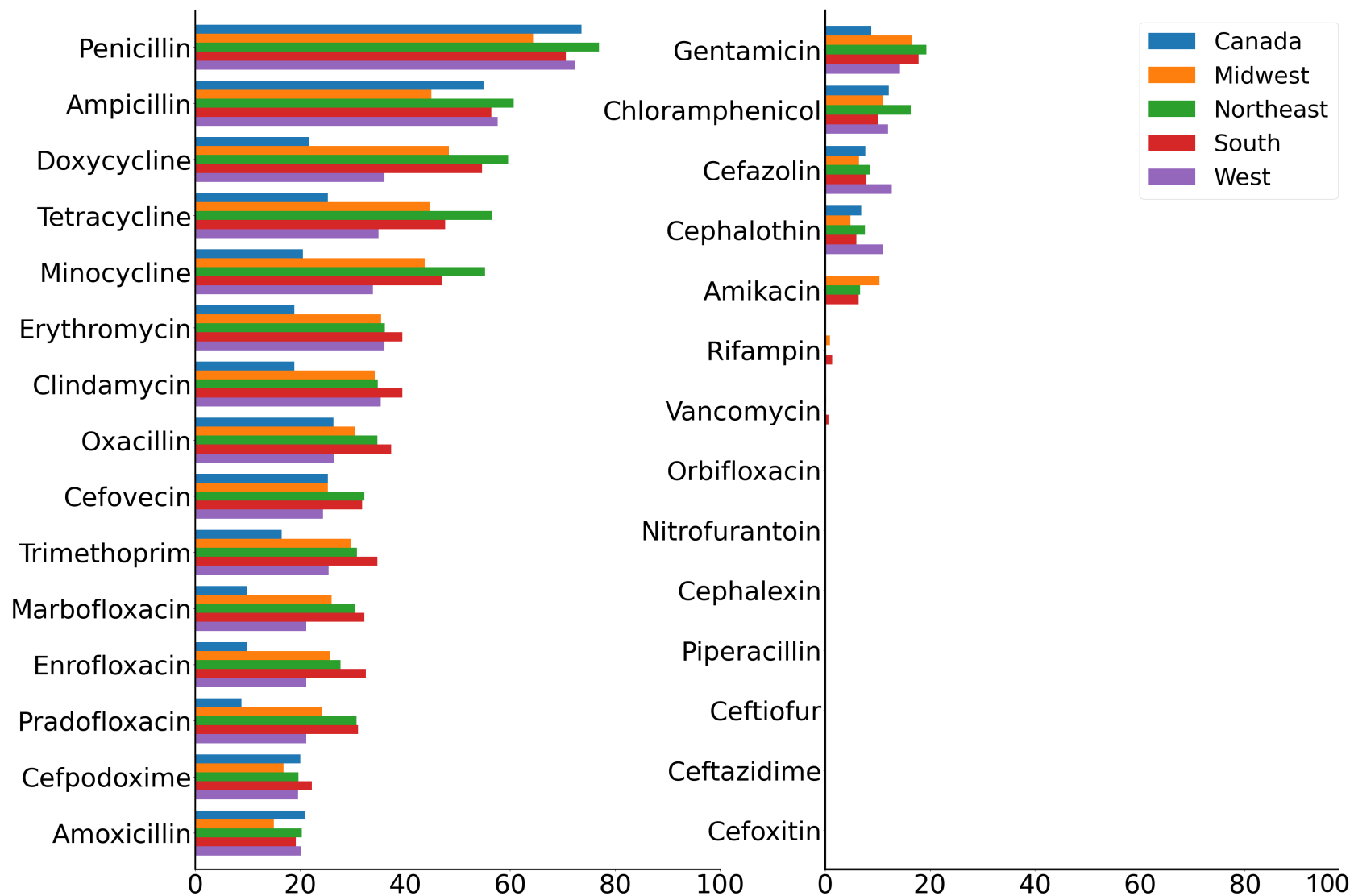

Supplement: Fig. S1 — Percentage of lab tested isolates resistant to various antibiotics across the geographic regions of North America. [file aem.00010-25-s0001.pdf]

A.

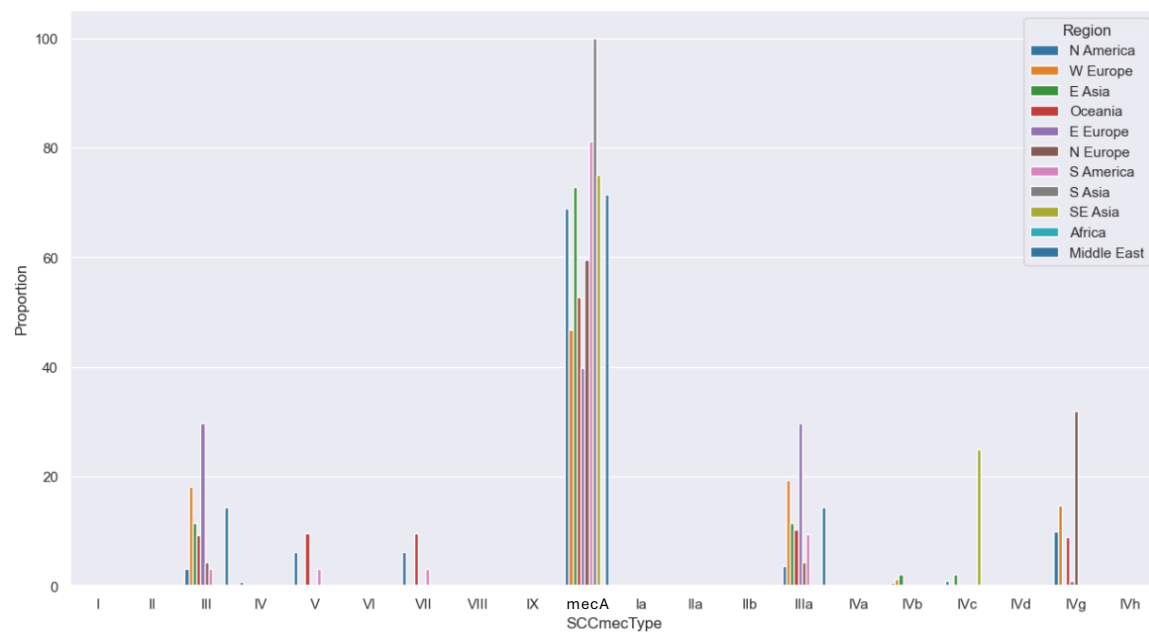

B.

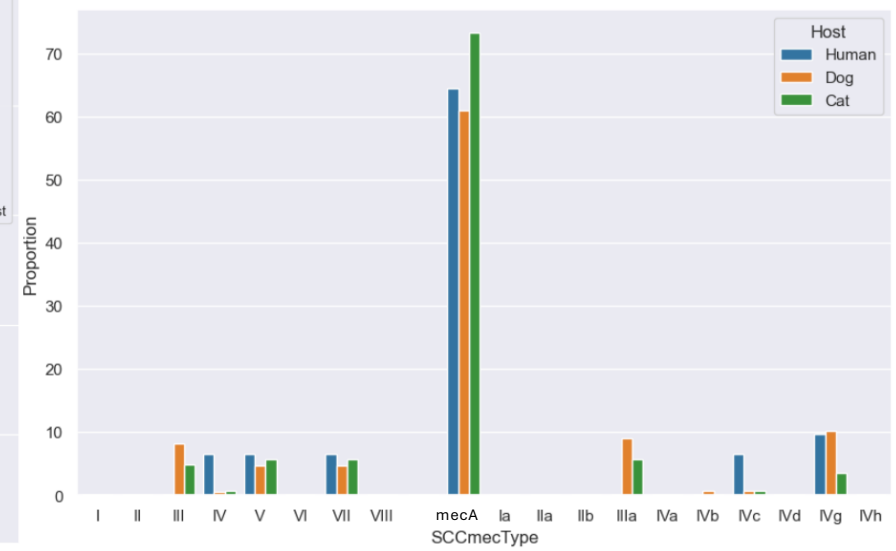

Supplement: Fig. S3 — Distribution of SCCmec types. [file aem.00010-25-s0003.pdf]

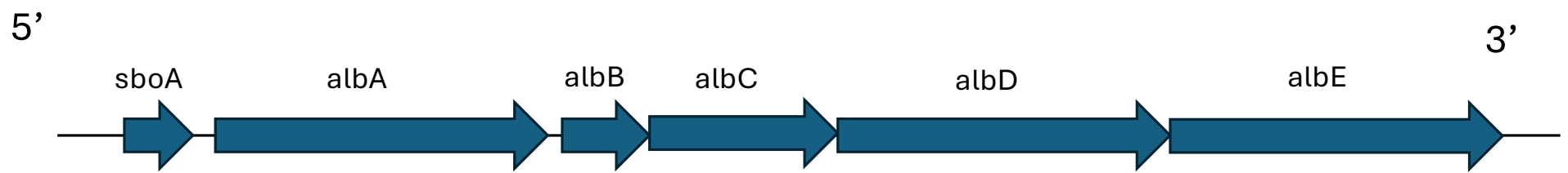

Supplement: Fig. S4 — sbo-alb operon. [file aem.00010-25-s0004.pdf]
