# Supplementary material for: Population and pan-genomic analyses of Staphylococcus pseudintermedius identify geographic distinctions in accessory gene content and novel loci associated with AMR
Source: Appl Environ Microbiol. 2025 Apr 24;91(5):e00010-25. doi: 10.1128/aem.00010-25 (PMC12094015; doi:10.1128/aem.00010-25)
Supplement: Fig. S2 — Genes that were the greatest contributors to separation of the PC2 axis in the principal component analysis of accessory gene content. [file aem.00010-25-s0002.pdf]

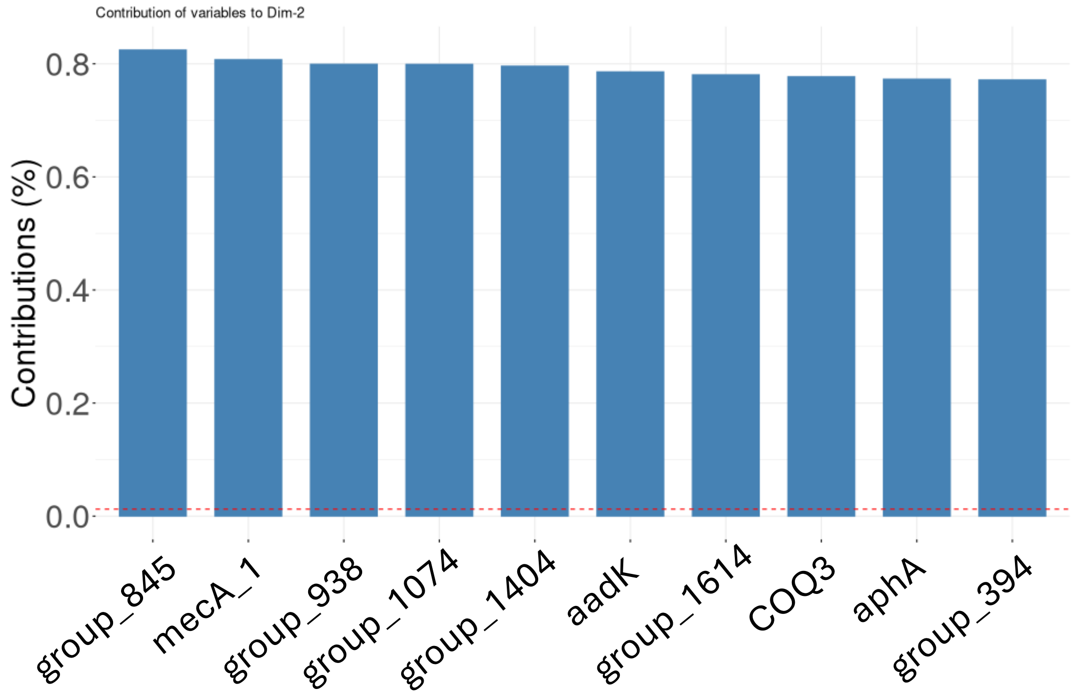

group_845	hypothetical protein
mecA_1	MecA
group_938	hypothetical protein
group_1074	hypothetical protein
group_1404	IS1182 family transposase IS1182
aadK	Aminoglycoside 6-adenylyltransferase
group_1614	hypothetical protein
COQ3	Ubiquinone biosynthesis O-methyltransferase
aphA	Aminoglycoside 3'-phosphotransferase
group_394	hypothetical protein
