# Supplementary material for: Population and pan-genomic analyses of Staphylococcus pseudintermedius identify geographic distinctions in accessory gene content and novel loci associated with AMR
Source: Appl Environ Microbiol. 2025 Apr 24;91(5):e00010-25. doi: 10.1128/aem.00010-25 (PMC12094015; doi:10.1128/aem.00010-25)
Supplement: Supplemental legends — Legends for all supplemental material. [file aem.00010-25-s0005.docx]

**Suppl. Fig Legends**

**Fig. 1.** Percentage of lab tested isolates resistant to various antibiotics across the geographic regions of North America.

**Fig. 2.** Genes that were the greatest contributors to separation of the PC2 axis in the principal component analysis of accessory gene content in Fig 4 of the main manuscript. The highlighted genes are resistance determining mechanisms, all but MecA linked to aminoglycoside resistance. All of these genes were more common in E. Asia versus North America and explain much of the separation between these two global regions on the PCA2 axis.

**Fig. 3.** SCCMec types: (A) global regions and (B) between cat and dog hosts of the North American isolates.

**Fig. 4.** *Staphylococcus pseudintermedius sboA* operon.

**Suppl. Table 1.** Number of isolates represented for each country used in this study.

**Suppl. Table 2.** Percentage of number of phenotypically resistant isolates containing a known AMR gene that is also Scoary significant by total number of phenotypically resistant isolates for each antibiotic tested.

**Suppl. Table 3.** Scoary significant genes for each antibiotic tested. All Scoary results are listed for each gene, along with indication if the gene is a known AMR gene (as determined by AMRFinderPlus – “known_AMR”), and if that gene is correlated with phenotypic resistance to multiple antibiotics (“Correlated_with_other_Antibiotics”).
